# The Structure of HasB Reveals a New Class of TonB Protein Fold

**DOI:** 10.1371/journal.pone.0058964

**Published:** 2013-03-19

**Authors:** Gisele Cardoso de Amorim, Ada Prochnicka-Chalufour, Philippe Delepelaire, Julien Lefèvre, Catherine Simenel, Cécile Wandersman, Muriel Delepierre, Nadia Izadi-Pruneyre

**Affiliations:** 1 Institut Pasteur, Unité de Résonance Magnétique Nucléaire des Biomolécules, Département de Biologie Structurale et Chimie, Paris, France; 2 CNRS, UMR 3528, Paris, France; 3 Institut de Biologie Physico-Chimique, CNRS Université Paris-Diderot UMR 7099, Paris, France; 4 Institut Pasteur, Unité des Membranes Bactériennes, Département de Microbiologie, Paris, France; La Trobe University, Australia

## Abstract

TonB is a key protein in active transport of essential nutrients like vitamin B12 and metal sources through the outer membrane transporters of Gram-negative bacteria. This inner membrane protein spans the periplasm, contacts the outer membrane receptor by its periplasmic domain and transduces energy from the cytoplasmic membrane pmf to the receptor allowing nutrient internalization. Whereas generally a single TonB protein allows the acquisition of several nutrients through their cognate receptor, in some species one particular TonB is dedicated to a specific system. Despite a considerable amount of data available, the molecular mechanism of TonB-dependent active transport is still poorly understood. In this work, we present a structural study of a TonB-like protein, HasB dedicated to the HasR receptor. HasR acquires heme either free or *via* an extracellular heme transporter, the hemophore HasA. Heme is used as an iron source by bacteria. We have solved the structure of the HasB periplasmic domain of *Serratia marcescens* and describe its interaction with a critical region of HasR. Some important differences are observed between HasB and TonB structures. The HasB fold reveals a new structural class of TonB-like proteins. Furthermore, we have identified the structural features that explain the functional specificity of HasB. These results give a new insight into the molecular mechanism of nutrient active transport through the bacterial outer membrane and present the first detailed structural study of a specific TonB-like protein and its interaction with the receptor.

## Introduction

In addition to a cytoplasmic membrane, which is common to all organisms, Gram-negative bacteria possess an outer membrane (OM) that acts as a selective permeation barrier. Indeed, a number of different transport pathways regulate the uptake of essential compounds into the cell. In contrast to small molecules that cross the outer membrane by passive or facilitated diffusion through transmembrane porins, nutrients that are either poorly permeable through porins (those greater than 600 Da) or present at very low concentration, are transported *via* outer membrane specific transporters by an energized process [1], [2]. The following substrates are internalized by this active transport process: vitamin B12, heme, iron-siderophore and nickel-nickelophore complexes and some carbohydrates [3]. No energy source is present at the OM, the energy for this process is provided by an inner membrane complex composed of TonB, ExbB and ExbD proteins [4], which couples the inner membrane proton-motive force (pmf) to the TonB-dependent outer membrane transporter (TBDT). These TBDTs share common structural features and are composed of a C-terminal β-barrel and a N-terminal globular domain, folding back into and closing, like a “plug”, the channel formed by the barrel [5]. TonB is composed of three domains. The N-terminal transmembrane helix anchors the protein to the inner membrane and makes contact with ExbB and ExbD to form an energy transducing complex. The C-terminal globular domain directly contacts the transporters in the OM. These two domains are separated by a flexible, unstructured proline-rich domain that resides within the periplasm. No three-dimensional (3D) structure is currently available for any entire TonB protein. However, several structures of the C-terminal domain of *E. coli* TonB, either free or complexed with a TBDT, have been solved by X-ray crystallography or NMR [5]. The TBDT-TonB structures show that TonB interacts with residues from the exposed periplasmic surface of the transporter. A critical region for this interaction involves a relatively conserved stretch of residues called the TonB box, which forms an inter-protein β-sheet with the third β-strand of TonB [6], [7]. The molecular mechanism of TonB-dependent active transport is still poorly understood. It is currently accepted that under energized TonB action, the receptor plug undergoes large conformational changes within the barrel to form a transient channel or to be pulled out of the barrel, either as a globular unit or in an unfolded state [8].

When, as in *E. coli*, only one TonB protein is presented in the bacterial genome, it is shared by different TBDTs involved in in the acquisition of various substrats. These TBDTs then compete for a limited amount of this unique TonB [9]. Nevertheless most of bacteria that have a variety of TBDTs involved in uptake of various nutrients in different conditions have more than one *tonB* gene, their number going from two to nine [10]. Some of them exhibit partially redundant function with distinct specificities. In the case of *Vibrio anguillarum*, among the two TonB systems, only TonB2 is involved in iron siderophore anguibactin transport. Recently, its C-terminal domain has been characterized and structurally studied. Two structural differences are observed between this domain and that of *E. coli* TonB: the loop 3 is extended by 9 Å in TonB2 and the last C-terminus β-strand of *E. coli* TonB is not seen in TonB2. The analysis of mutant proteins has shown that these structural differences do not completely explain the functional specificity of TonB2 for ferri-anguibactin TBDT [11].

*Serratia marcescens* genome analysis has shown the presence of several *tonB-exbB-exbD* genes, six *tonB* paralogs and two *exbB-exbD* pairs [12], [13]. One of them, *hasB,* unlinked to any *exbB-exbD* genes, is located in the *has* operon [14]. This operon is negatively regulated by iron *via* the Fur protein and positively regulated by a sigma/anti-sigma signaling cascade [15]. The proteins encoded by the *has* operon belong to the Has system which is found in several Gram-negative bacteria and allows the internalization of heme for iron utilization; heme being a major iron source for pathogenic or commensal bacteria in mammals. This system involves an extracellullar protein, the HasA hemophore that captures free or hemoprotein-bound heme and delivers it to a specific outer membrane transporter (HasR). While neither the interaction of the transporter with heme and hemophore nor the transfer of heme from the hemophore to the transporter require energy, the ensuing steps of heme internalization through the transporter and ejection of the empty hemophore consume energy provided by HasB [16], [17].

HasR belongs to the sub-family of the TBDT having, in addition to their transporter role, a signaling activity [18]. The presence of the ligands on the extracellular face of these transporters is signaled through their N-terminal signaling domain located on their periplasmic face, to a sigma factor regulator. The latter, also called anti-sigma factor, sends then this signal to a sigma factor inducing the transcription of the relevant genes. Although the structure of HasR complexed either with HasA and heme or with HasA alone is known, neither the TonB box nor the signaling domain are seen in these structures [19].

HasB shares about 20% of sequence identity with both TonB of *S. marcescens* and *E. coli*. Sequence analysis of HasB reveals the same three-domain structural organization as that of TonB. Both HasB and TonB are functional with HasR, either for transport activity or for signaling. However HasB is specific for this receptor and more efficient than TonB, in both *S. marcescens* and a reconstituted system in *E. coli* using *E. coli* TonB [14].

In order to determine the basis of the specificity of HasB for HasR, we studied, in a previous work [20], the *in vitro* interaction of HasR with HasB_CTD_ (HasB C-terminal domain)_,_ a periplasmic fragment of HasB (residues 133–263, formerly named HasB_133_) and compared it with that of an equivalent domain of *E. coli* TonB (TonB_CTD_, previously named TonB_116_). We have demonstrated that while TonB_CTD_ (TonB C-terminal domain) behaves as a “generalist energy transducer” interacting within the same range of affinity and with wide range of TBDTs, the HasB-HasR interaction presents a higher affinity (K_d_ = 13 nM) and a different interaction network [20]. Having observed the difference of binding mode between HasB and TonB, we wondered whether it is due to structural differences between the two proteins and/or to the fact that they do not recognize the same region of HasR.

In this work, we solved the solution structure of HasB_CTD_ using heteronuclear NMR. The structure reveals significant differences compared to known structures of TonB and is therefore representative of a new structural class of the TonB proteins.

To gain more insight into the mechanism of active transport *via* a specific energy transducer, we studied the interaction of HasB_CTD_ with a peptide corresponding to the TonB box of the HasR transporter, since the TonB box is believed to be the main region of protein-protein interaction. On the basis of our structural data and a docking model of the complex between HasB and the HasR TonB box, two mutants of HasB have been constructed for further investigation of the interaction between these two partners.

## Materials and Methods

### Protein Purification

Labeled and unlabeled HasB_CTD_ (named HasB_133_ in our earlier publication [20]) were prepared as described [21]. TonB_CTD_ of *E. coli*_,_ previously named TonB_116_, was prepared as already reported [20]. HasB mutations were introduced by PCR into the plasmid encoding HasB_CTD_ wild type with the following pairs of oligonucleotides:

R38A:GGGGAGGACAACTGGGCCAGCCGCATCAGC;

R38Ar: GCTGATGCGGCTGGCCCAGTTGTCCTCCCC;

R38E: GGGGAGGACAACTGGGAAAGCCGCATCAGC;

R38Er: GCTGATGCGGCTTTCCCAGTTGTCCTCCCC; mutations were verified by sequencing.

The TonB box peptides were synthesized with free acid C- and amine N-termini by *Proteogenix* (Strasbourg, France).

### NMR Spectroscopy

All NMR experiments were recorded at 293K on a Varian 600 MHz spectrometer with a cryogenically cooled triple resonance ^1^H/(^15^N/^13^C) PFG probe. Proton chemical shifts were referenced to 2,2-dimethyl-2-silapentane-5-sulfonate (DSS) as 0 ppm. ^15^N and ^13^C chemical shifts were referenced indirectly to DSS. The pulse sequences were taken as implemented in the Varian BioPack. NMR data were processed using NMRPipe/NMRDraw [22] and spectra were analyzed using NMRViewJ and CCPNMR Analysis [23], [24]. The HasB samples used for NMR experiments were from 0.35 to 0.7 mM in 50 mM sodium phosphate buffer, pH 7, 50 mM NaCl and 15% D_2_O/H_2_O (v/v).

HasB mutants NMR samples were 0.2 mM in the same buffer.

### Structure Calculation

HasB_CTD_ structures were calculated using 2117 NOE-derived distance constraints, 148 dihedral angles and 23 hydrogen bonds. Distance constraints were obtained from 3D ^13^C and ^15^N NOESY-HSQC experiments with mixing times of 100 and 80 ms, respectively. Dihedral angle restraints were determined using TALOS+ program [25], [26]. Hydrogen bond donors were obtained from a series of ^1^H-^15^N HSQC experiments of lyophilized HasB_CTD_ dissolved in ^2^H_2_O and monitored for 36 hours. The corresponding hydrogen bond acceptors were identified based on the NOE pattern from the 3D HSQC-NOESY experiments.

Structure calculation was combined with automatic NOE cross-peak assignment using ARIA version 2.2 and CNS version 1.2 [27], [28]. Several cycles of ARIA were performed using standard protocols with spin diffusion corrections. After each cycle rejected restraints, assignments and violations were analyzed. Finally, 500 conformers were calculated with ARIA/CNS, and the 20 water refined structures with the lowest restraint energy values were used for statistical analysis. The structures were visualized and analyzed with MOLMOL [29] and PYMOL (DeLano, W.L., Copyright 2009–2010 Schrödinger LLC), their quality was assessed using PROCHECK [30] and WHATCHECK [31].

### NMR Backbone Dynamics, Data Acquisition and Analysis

One set of T_1_ and T_2_ experiments and two sets of steady-state^1^H-^15^N NOE were recorded on a Varian Inova 500 MHz spectrometer equipped with a triple resonance z-pulse field gradient probe using standard Varian BioPack pulse sequences. For T_1_ experiments, relaxation delays were 0.02, 0.06, 0.26, 0.50, 1.01, 1.20, 1.50 and 2.00 s, and for T_2_ experiments, 0.01, 0.03, 0.05, 0.07, 0.09, 0.13, 0.15 and 0.17 s. The analysis was done using the Rate Analysis package in NMRViewJ. The R_1_ and R_2_ relaxation rates were determined by fitting the T_1_ and T_2_ peak intensities to a single-exponential decay. NOE values are the ratios between the intensities of corresponding peaks in the spectra recorded with and without pre-saturation of the amide protons. The HasB_CTD_ sample used for relaxation experiments was 0.35 mM in 50 mM sodium phosphate buffer, pH 7, 50 mM NaCl and 10% D_2_O/H_2_O (v/v).

### Chemical Shift Perturbation (CSP) Analysis

CSP values of ^1^H and ^15^N amide of HasB_CTD_ were obtained after interaction with the TonB box peptide by comparing the ^1^H-^15^N HSQC spectra of free and bound HasB_CTD_ as previously explained [32]. The samples were prepared in 50 mM sodium phosphate buffer, pH 7, 50 mM NaCl and 15% D_2_O/H_2_0 (v/v). Lyophilized peptides were added to the HasB_CTD_ solution at 1∶2, 1∶1, 2∶1 and 4∶1 ratios. CSP values greater than 0.2 ppm were considered as large. For proline residues no chemical shift change can be observed because of the lack of backbone amide proton.

### Peptide Resonance Assignment

2D ^13^C/^15^N-filtered NOESY and TOCSY spectra (Varian BioPack version 2008-05-01) of the TonB box peptide were acquired in the presence of ^15^N-^13^C labeled HasB_CTD_. In these experiments only cross-peaks between protons attached to ^12^C carbons are observed. They efficiently filter all protein resonances and allow for the straightforward assignment of bound peptide resonances.

### Intermolecular NOEs

For selectively displaying NOE cross peaks originating from ^12^C- and ^14^N- bound protons from the peptide, we performed 3D ^13^C/^15^N F1-filtered, F3-edited NOESY-HSQC experiments [33]. The spectrum displays exclusively NOE peaks between peptide ^1^H resonances (along the F3 dimension) and protein ^1^H-^13^C/^15^N group resonances (along the F1 (^1^H) and F2 (^13^C/^15^N) dimensions). Three intermolecular NOEs were observed in the spectrum and subsequently used as distance constraints in the calculation of the structural model of the complex between HasB_CTD_ and the TonB box peptide. These NOEs secure the correct orientation of the members of the complex.

### Docking of the Complex Structure

A model of the complex of HasB_CTD_ with the 21-mer peptide was built using the standard protocols of program HADDOCK, version 2.0 [34]. Initial coordinates of HasB_CTD_ in the free form were taken from our PDB entry 2M2K whereas the free peptide was defined in extended conformation. Twenty-three ambiguous interaction restraints, defined on the basis of the CSP and surface accessibility analysis were used as the docking driving information together with three intermolecular NOEs, which also assured the correct orientation of the peptide relative to HasR. Two hundred complex structures were generated and refined in water. Twenty structures with lowest energy values were then analyzed. The lowest energy structure of the complex, representative of this family of structure, was chosen as the model of the complex shown here.

### Isothermal Titration Calorimetry (ITC)

ITC was performed at 25°C using a MicroCal VP titration calorimeter (MicroCal-GE Healthcare) under the same conditions as described previously [20]. The buffer was 20 mM sodium phosphate, pH 7, 50 mM NaCl for all the titrations, except for experiments carried out in the presence of HasR where 20 mM sodium phosphate, pH 7, 0.08% ZW 3–14 was used.

The heat of dilution of peptide/protein injections was determined either by injecting the ligand into the buffer alone or by injecting the ligand into the cell after the saturation of the protein binding site. The value obtained was subtracted from the heat of reaction to give the effective heat of binding. The resulting titration data were analyzed using Microcal-ORIGIN software package. The molar binding stoichiometry (N), association constant (K_a_; K_d_ = 1/K_a_) and enthalpy changes (ΔH) of binding were determined by fitting the binding isotherm to a model with one set of sites. Their values are the average of three experiments. HasB_CTD_/TonB_CTD_ titrations with peptides were carried out by injecting 20–35 consecutive aliquots of 5–10 µL of a 1–1.5 mM peptide solution into the ITC cell containing 4 to 9×10^−5^ M of protein solution of either HasB_CTD_ or TonB_CTD_. For HasR titration experiments with HasB, 5–8×10^−5^ M of HasB_CTD_ wild type or mutants and 3–5 µM of HasR were used.

## Results

### Structure and Dynamics of HasB_CTD_

**Figure 1:**
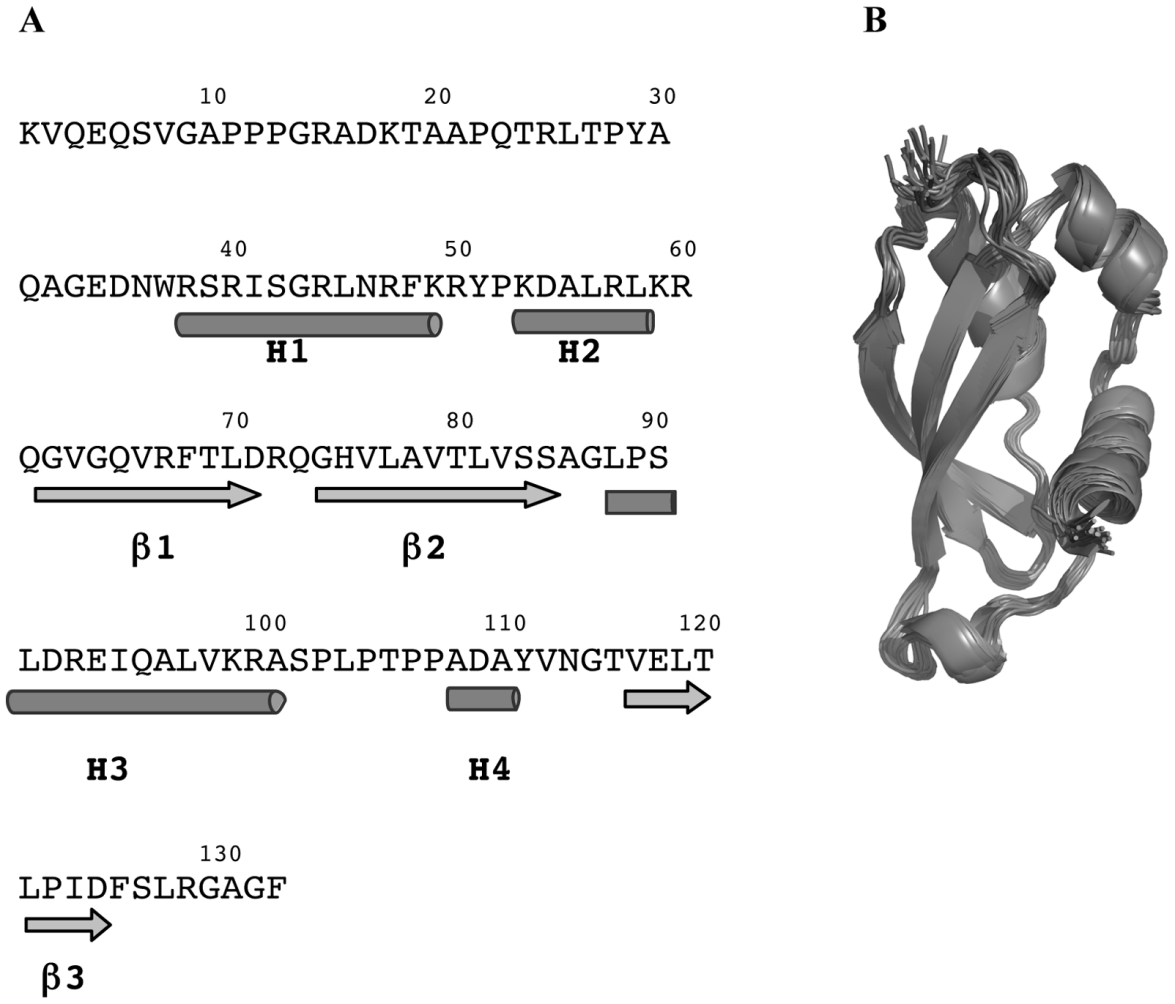
Overall structure of HasB_CTD_. A: Secondary structure elements in HasB_CTD_. Arrows represent the β-strands and cylinders the α-helices. B: Ribbon diagram of backbone superposition of the 20 final structures of HasB_CTD._ The N-terminal tail (residues 1–35) is not presented.

The solution structure of HasB_CTD_ exhibits three distinctive regions: the N-terminal tail composed of the first 35 residues, followed by the globular folded region (residues 36–127) and an unstructured C-terminal region comprising the residues 128–132 (Fig. 1A and B).

The structured region consists of a three-stranded anti-parallel β-sheet and three α-helices, located on the opposite sides of the protein. The two sides are separated by a long loop containing an additional short α-helix H4.

The details of the constraints and structural characteristics of the family of 20 conformers representing the solution structure of HasB_CTD_ are summarized in Table 1. The structures display a good convergence, with a mean pairwise root-mean-squared deviation (rmsd) of 0.94 and 1.59 Å for the backbone and heavy atoms respectively.

**Table 1:** Summary of the NMR constraints used for the structure calculation, the restraint violations and structural statistics for the ensemble of 20 best conformers of HasB_CTD._

NOE-derived constraints:	2117	Energies (kcal/mol)	
unambiguous:	1493	E_noe_	322.63±22.46
intraresidue	533	E_bond_	41.26±1.74
sequential	367	E_angle_	203.99±10.53
medium range	194	E_vdv_	−350.20±21.44
long range	399		
ambiguous:	624	Mean pairwise RMSD (Å) residues 36–126:	
intraresidue	74.1	Backbone atoms:	0.94±0.23
sequential	120.8	All heavy atoms:	1.59±0.27
medium range	109.2		
long range	319.9	Ensemble Ramachandran plot residues 36–126:	
Hydrogen bonds:	23	most favoured	84.6%
Talos (φ,ψ):	148	additional allowed	12.8%
Total number of experimental. restraints	2288	generously allowed	2.6%
Residual distance constraint violations:		disallowed	0.0%
> = 0.5 Å	1.70±0.70		
> = 0.3 Å	5.95±0.87		
RMS from NOEs (Å)	0.064±0.003		

**Figure 2:**
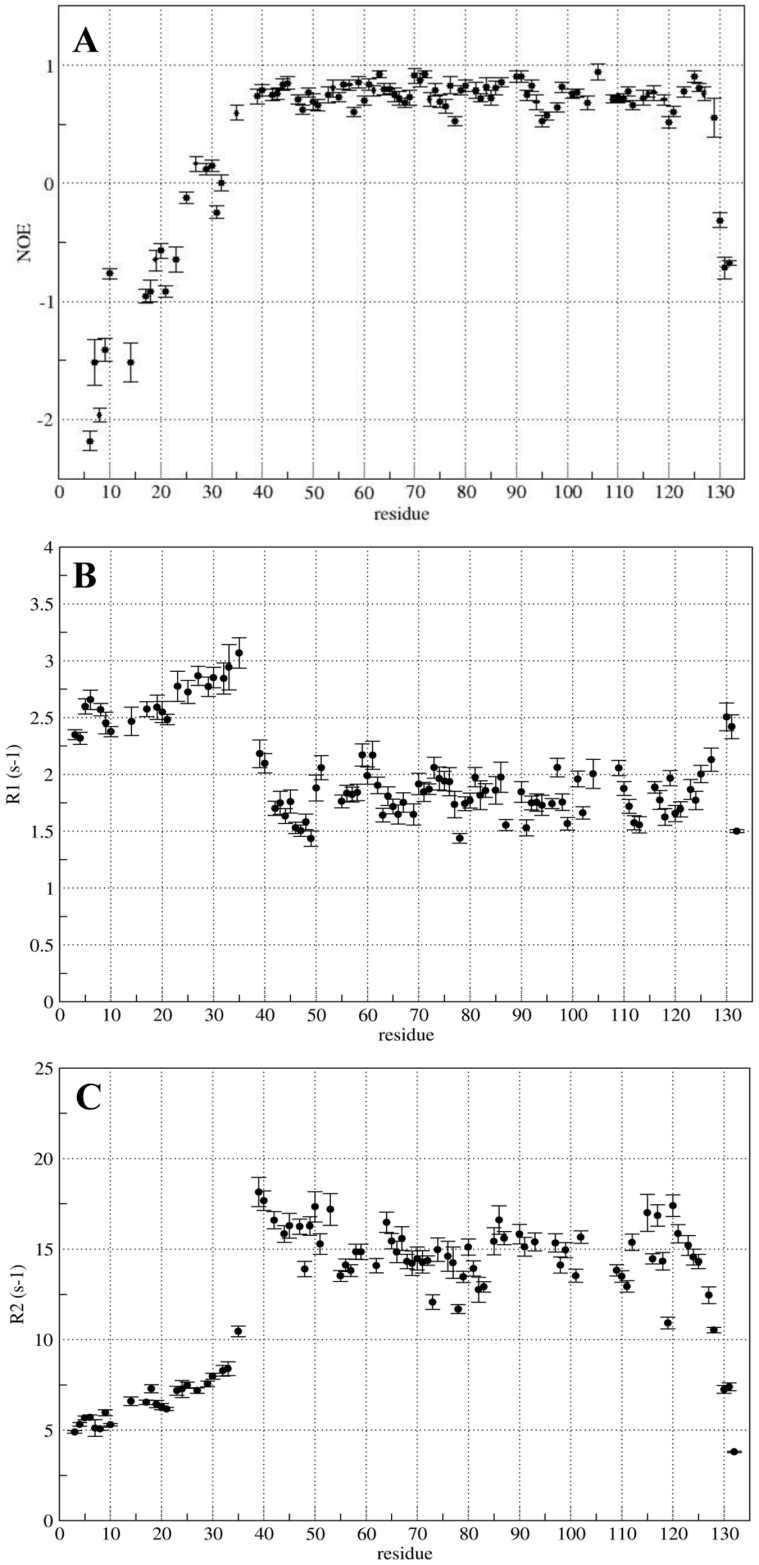
Backbone dynamics of HasB_CTD_ that identifies regions of well-defined structure and mobility. Steady-state ^15^N-^1^H NOE (A), relaxation rates R1 (B) and R2 (C) were measured at 500 MHz and 293 K.

The dynamic properties of the protein backbone were studied by measuring ^15^N longitudinal (R1) and transverse (R2) relaxation rates, as well as steady-state^15^N-^1^H NOE values. Their analysis shows a clear difference between the dynamical properties of the core domain and those of the N-terminal region (residues K2-D35). As can be seen in Figure 2A and 2B, the values of R1 are higher, and those of R2 are smaller than their respective average values. ^1^H-^15^N NOEs, very sensitive to the fast internal dynamics, remain negative for K2-D35 region (Fig. 2C) indicating that this portion of the protein is flexible and unstructured. On the other hand, the values of R1, R2 and the NOEs for the residues N36 to F127 are representative of a well-structured region with low flexibility and slow internal motions.

The atomic coordinates of the ensemble of 20 lowest energy conformers of HasB_CTD_ have been deposited in RCSB Protein Data Bank *(* http://www.rcsb.org *)* under the accession code 2M2K.

### HasB_CTD_ Interaction with HasR TonB Box Peptide

In order to define how HasB_CTD_ interacts with the TonB box of HasR, we studied the interaction of HasB_CTD_ with two peptides encompassing the putative TonB box (underlined) of HasR: a dodecamer (A^98^LDSLTVLGAGG^109^) and a 21-mer (G^92^DGGALALDSLTVLGAGGNNA^112^).

Isothermal titration calorimetry (ITC) and chemical shift perturbation (CSP) analysis were used to probe interaction between HasB_CTD_ and peptides. CSP is the most widely used NMR method to map protein interfaces. The interaction causes environmental changes in the residues located on the interacting surface and hence affects the chemical shifts of their backbone amides. This method is very sensitive to even subtle effects and allows observing the formation of a complex, even a transient one.

For the dodecamer peptide we did not observe any enthalpic signal of interaction with HasB_CTD_ by ITC. Moreover, no chemical shift perturbation could be observed in the ^15^N-^1^H HSQC spectra of HasB_CTD_ confirming the absence of interaction for this peptide, at least in the range of concentration used (up to 8×10^−4 ^M of protein and peptide/protein ratio of about 4∶1). However, the peptide interacts with TonB_CTD_ with an affinity of about 2×10^4^ M^−1^ (K_d_ = 50 µM, Fig. S1).

**Figure 3:**
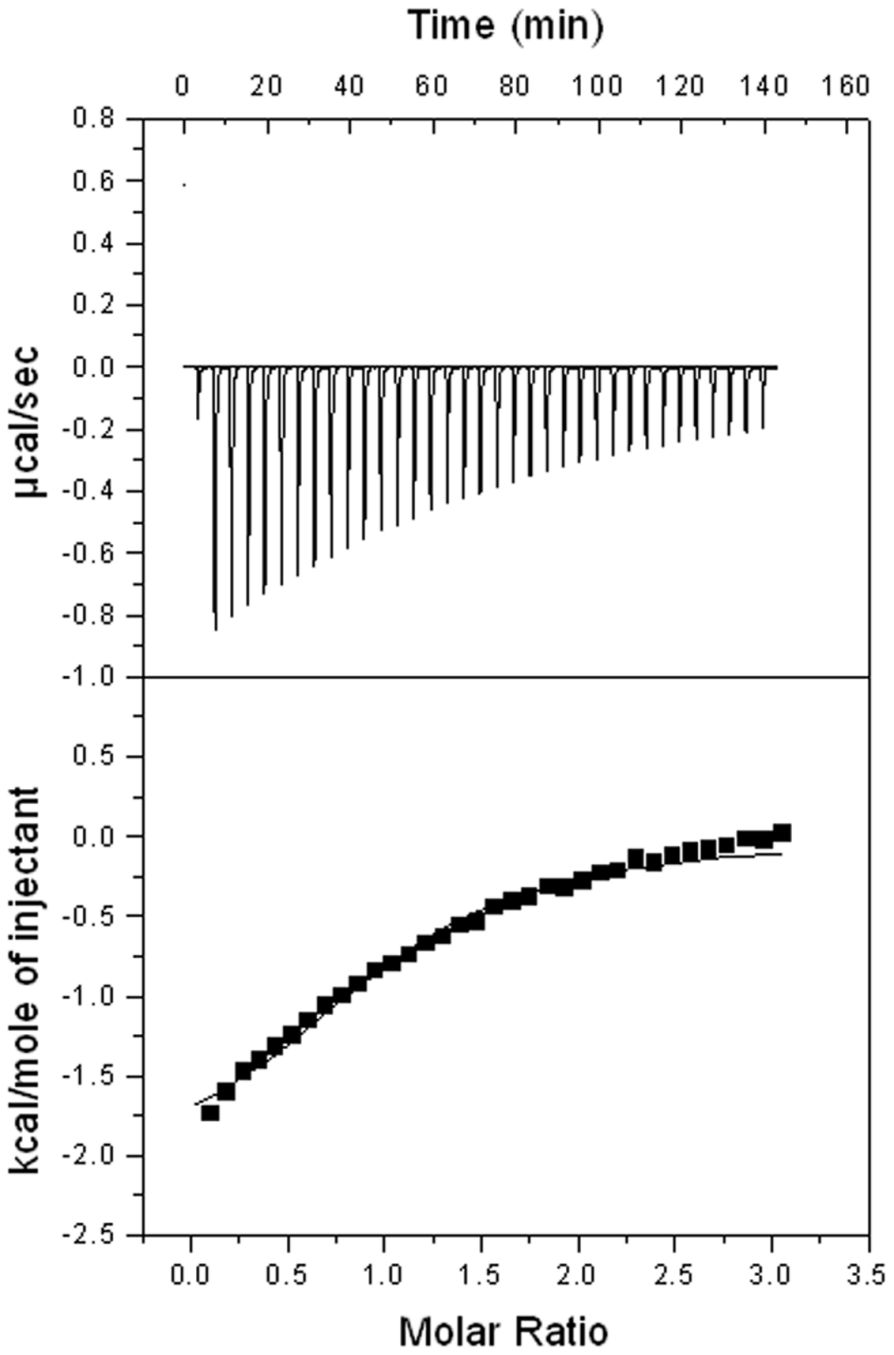
ITC analysis of the interaction of HasB_CTD_ with the 21-mer peptide corresponding to HasR TonB box. A representative experiment is shown. The heat signal (top) is presented together with the binding isotherm derived from the signal (bottom). The concentration of HasB_CTD_ and the peptide were respectively, 9×10^−5^ M and 1.5 mM.

The absence of interaction between HasB_CTD_ and the above mentioned peptide led us to test a longer peptide of 21 residues, exceeding the region equivalent to the first peptide, and corresponding to the region of HasR located between the N-terminal signaling domain and the structured part of the plug. This region is predicted to be unstructured and indeed, it is not seen in the electron density map of the X-ray structure of HasR as it is the case for the equivalent region of many TBDTs [19], [5]. Using 2D TOCSY, NOESY and ROESY experiments, we verified that the 21mer peptide was unstructured in solution (data not shown). Interestingly, in the presence of this peptide, several CSP were observed in the ^15^N-^1^H HSQC spectrum of HasB_CTD_ indicating that interaction occurs. The affinity constant of HasB_CTD_ for the 21-mer peptide of HasR determined by ITC is 4×10^4^ M^−1^ (K_d_ = 25 µM) (Fig. 3).

### Structural Analysis of the Complex HasB_CTD_ –21-mer TonB Box Peptide

**Figure 4:**
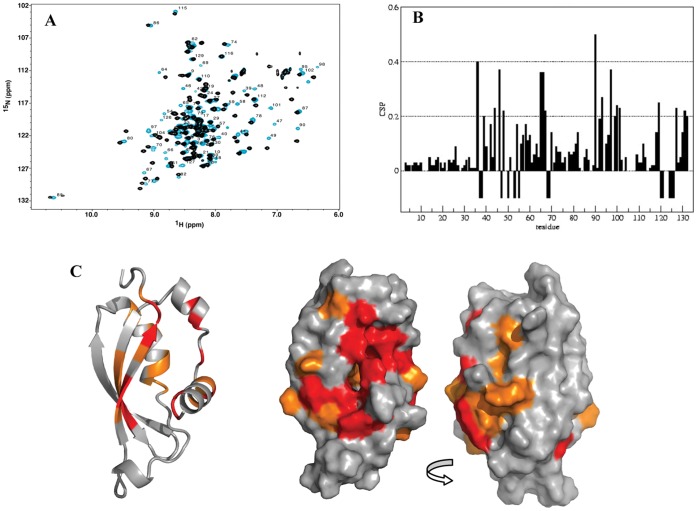
Analysis of HasB_CTD_ upon HasR TonB box peptide binding. A: ^15^N HSQC spectra of HasB_CTD_ in the presence (blue) or absence (black) of the TonB box peptide. B: CSP values shown by residue; C: CSP mapped onto HasB_CTD_ structure. Residues showing the greatest backbone amide shifts (>0.2 ppm) are shown in orange. Residues not seen in the ^15^N HSQC spectrum, due to intermediate chemical exchange, are indicated by negative bars in B and colored in red in C.

Due to slow exchange on the chemical shift time scale between the free and bound states of HasB_CTD_ it was not possible to infer the resonance assignments of the bound form from those of the free state. Therefore, the assignment of the backbone of the bound form of HasB_CTD_ was made using a set of classical 3D NMR experiments. The backbone amides of 12 out of 119 residues could not be assigned (Fig. 4A). Nevertheless, except for four residues (F68, L121, D124 and F125) for which none of the atoms could be assigned, structural information has been obtained for the remaining residues through their C and H atoms.

The analysis of the carbon (Cα, Cβ and CO) and Hα chemical shift values showed that the regular secondary structure elements of HasB_CTD_ were conserved between its free and bound forms. Furthermore, NOE patterns obtained for both forms did not display significant changes also suggesting that the overall fold of HasB_CTD_ is not changed upon the binding of the TonB box peptide.

In order to identify the region of HasB_CTD_ involved in formation of the complex with the 21-mer peptide, we have analyzed the chemical shift perturbations presented in Figure 4B. Since the overall fold of the HasB_CTD_ is maintained, the CSP reflect the interaction between the two molecules. They reveal that the most substantial changes observed upon peptide binding are located on the same side of HasB_CTD,_ especially along the strand β3 and on the helix H1 as shown in Figure 4C.

The CSP analysis only allows to localize the overall binding surface but it still remains unknown how the partners interact on an atom-to-atom basis. One approach to answer this question consists in combining the NMR shift mapping with a few distance constraints obtained from intermolecular NOEs. In our case, some of HasB_CTD_ residues in the interface region were in the intermediate chemical exchange regime and thus were not seen in the spectra. Consequently, the number of distance constraints between HasB_CTD_ and the peptide was very limited. Three distance constraints were used as selection filter in the docking program HADDOCK [34] in combination with twenty-three NMR defined ambiguous interaction restraints to calculate the HasB_CTD_-TonB box complex model.

**Figure 5:**
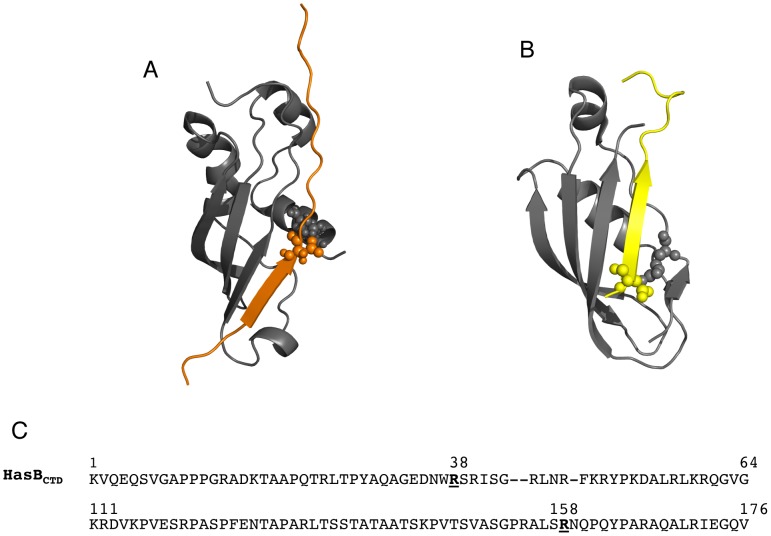
TonB box interaction with HasB_CTD_ and TonB_CTD_. The model of the complex HasB_CTD_-TonB box (A) is compared to the structure of the complex TonB_CTD_-TonB box (B). The TonB box of HasR is in orange, the BtuB TonB box (PDB code: 2GSK) is in yellow. Intermolecular ionic interactions are shown in spheres. The residues of HasB_CTD_ and TonB_CTD_ involved in the intermolecular ionic interaction with TonB box are indicated in bold (C).

In the model of the complex HasB_CTD_-TonB box peptide, HasB_CTD_ conserves its overall structure. The peptide is bound parallel to the strand β3 of HasB_CTD_. The N-terminal part of the peptide between positions 95 and 100 (G_95_ALALD_100_) forms an additional strand β4, facing the stretch of β3 residues 117–120, hence contributing to the formation of an intermolecular four-stranded β-sheet. Further analysis of hydrogen bonding suggests that the strand β4 can even be longer, stretching up to the peptide residue L102 (hydrogen bonded to HasB_CTD_ P122). Most of the HasB_CTD_ residues interacting with the peptide belong to the strand β3 and to the α-helix H1 of HasB_CTD_ although the side chains of the helix H2 are also involved in the interaction. An intermolecular ionic interaction is formed between the side chains of the residues R38 of HasB_CTD_ and D100 of the peptide (Fig. 5A).

### Key Residues of the Interaction between HasB and HasR

**Figure 6:**
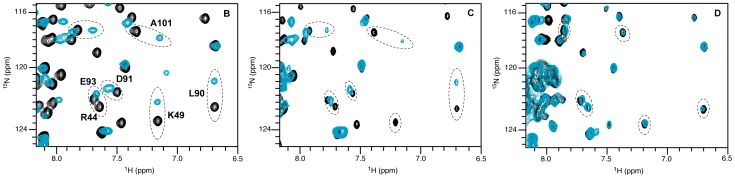
^1^H-^15^N HSQC spectra of HasB_CTD_ wild type (A), HasBR38A (B) and HasBR38E (C) in the presence (blue) or absence (black) of the TonB box peptide. Some of the residues experiencing chemical shift perturbation upon peptide binding in HasB_CTD_ wild type (A) and HasBR38A (B) are circled with dashed lines. The equivalent residues are indicated for HasBR38E (C) with no chemical shift changes.

The residue R38 of the α-helix H1 is viewed by our model as important for the interaction between HasB_CTD_ and the TonB box peptide (Fig. 5A). In order to confirm the role of this residue, we have produced two mutant proteins in which R38 was replaced either by alanine (HasBR38A) or by glutamic acid (HasBR38E). These two mutations do not modify the overall fold of HasB as verified by their NMR spectral signatures (data not shown). The analysis of their interaction with the TonB box peptide by NMR reveals that whereas HasBR38A is still able to interact with the peptide, the replacement of R by E abolishes this interaction (Fig. 6A, B and C).

In order to evaluate the effect of these mutations on the interaction between HasB and HasR and the contribution of R38 to this interaction, we have analyzed the interaction of each of the mutants with the entire HasR by ITC. This method allows to characterize molecular interactions, to measure their thermodynamic parameters and thus evaluate the contribution of polar interactions in the complex formation.

**Figure 7:**
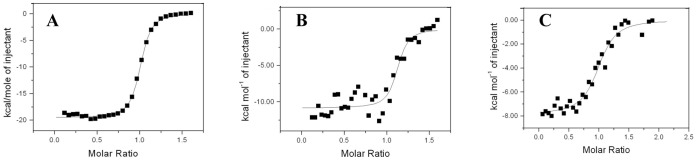
ITC titration curve of HasR with A: wild type HasB_CTD_ (ΔH = −19.5 kcal.mol ^−**1**^ **= −81.6 kJ.mol** ^−**1**^ **) B: HasBR38A (ΔH = −10.9 kcal.mol** ^−**1**^ **= −45.6 kJ.mol** ^−**1**^ **and C: HasBR38E (ΔH = −7.6 kcal.mol** ^−**1**^ **= −31.8 kJ.mol** ^−**1**^ **).** Representative experiments are shown.

The ITC titration curves of these mutants with the entire HasR are presented in Figure 7. The mutation affects the thermodynamic parameters of interaction between each mutant and HasR. The ΔH value representing polar interactions is highly modified for the titration of each of the mutants with HasR. Indeed, their ΔH value, −10.86 and −7.84 kcal.mol^−1^ (or −45.45 and –32.81 kJ. mol^−1^) for HasBR38A and HasBR38E respectively, are less negative compared to those obtained in our previous work [20] for the interaction between HasB_CTD_ wild type and HasR (ΔH = −19.5 kcal. mol^−1^ = −80.9 kJ. mol^−1^) and presented in Figure 7 A.

## Discussion

**Figure 8:**
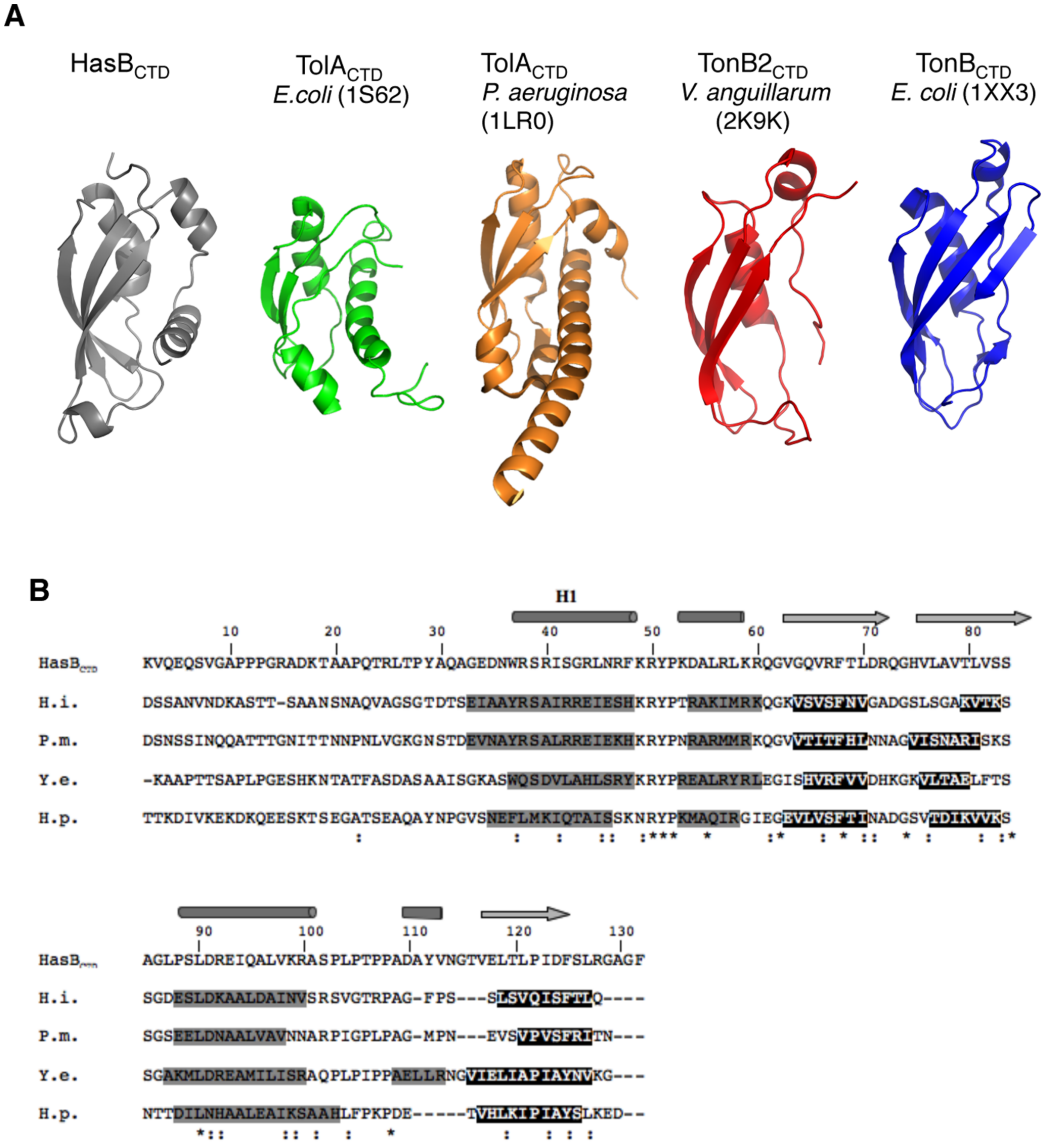
Structural comparison of HasB_CTD_ with TolA_CTD_ and TonB_CTD_. A: Ribbon representation of available solution structure of TonB and TolA proteins family. **B:** ClustalW alignments of HasB_CTD_ and TonB_CTD_ with their corresponding secondary structure. Cylinders represent the helices and arrows the β-strands of HasB_CTD_ structure. The secondary structure elements of other proteins are from a consensus prediction generated by NPS@ (Combet C, Blanchet C, Geourjon C, & Deléage, G (2000). NPS@: network protein sequence analysis. *Trends. Biochem. Sci.* **25**, 147–150). Predicted helices and β-strands are respectively presented in black on gray background and in white on black background. (*) conserved residues; (:) similar residues. The sequence numbering is that of HasB_CTD._ H.i.: residues 143–264 of *Haemophilus influenzae* TonB (#68248858); P.m.: residues 133–256 of *Pasteurella multocida* TonB (#33318341); Y.e.: residues 133–256 of *Yersinia enterocolitica* TonB (#A1JIK8); H.p.: residues 155–279 of *Helicobacter pylori* TonB1341 (#889856).

We have solved the 3D structure of HasB_CTD_ by NMR. The core of this domain (residues 36–132) adopts an ααββααβ fold, while residues 2–35 are flexible and unstructured. This 3D structure exhibits several differences from known structures of TonB (Fig. 8A), the most important being the presence of an additional N-terminal α–helix. This helix is predicted not only for all HasB proteins, but also for several TonB proteins (Fig. 8B). This finding leads to the conclusion that HasB represents a new structural class of TonB proteins. Some of them, like HasB or TonB1341 of *Helicobacter pylori* specific for nickel acquisition [3], are dedicated to a particular transporter. Others, like TonB of *Pasteurella multocida* or *Haemophilus influenzae,* are unique TonB of the bacterium and have then to be shared by various receptors. Therefore, it seems likely that the specificity of a TonB protein for a given transporter may not only be defined by a structural motif of TonB, but may also depend on the interacting regions of the transporter. The HasB_CTD_ β-sheet contains three β strands, whereas a supplementary strand is observed in the *E. coli* TonB_CTD_ (Fig. 8A). However, this strand is neither present in the known structure of *V. anguillarium* TonB_2CTD_ nor predicted for the majority of TonB proteins. The presence of this strand seems to be a particularity of the *E. coli* TonB [10].

An unexpected feature of HasB_CTD_ appeared when we submitted its 3D structure to Dali server [35] in order to find its possible structural neighbors among the known protein structures. HasB_CTD_ displays more structural similarity with TolA_CTD_ (Z score of 5.2 over 85 aligned residues) than with TonB proteins (Z score of 4.2 over 69 aligned residues) despite sharing only 11% of sequence identity with the former and 17% with the latter. Comparison of the topology of HasB, TolA and TonB CTDs is presented in Figure 8A and S2. TolA is the central protein of Tol-Pal complex. This system plays important roles in maintaining outer membrane integrity, transmembrane transportation, and cell division in Gram-negative bacteria [36], [37]. TolA shares many characteristics with TonB. It forms a complex with the TolQ and TolR inner membrane proteins, which display sequence similarity with ExbBD and MotAB of the bacterial flagellum [38]. Although the cumulative evidence indicates that TolA, like TonB, is an energy transducer, each of them possesses its own energy-harvesting complex, either ExbBD or TolQR, which can be imperfectly replaced by the other [39]. The structural similarity between TolA and HasB CTDs raised the question about the identity of the energy-harvesting complex of HasB, not yet known. It appears that ExbBD are the partner of HasB (P. Delepelaire, data to be published). HasB is the first example of a TonB like protein more similar structurally to TolA than to TonB and having ExbBD as partners.

The first 35 residues of HasB_CTD_ are unstructured and the average length of this segment is around 45 Å in the ensemble of 20 conformers representing the solution structure of HasB_CTD._ This segment makes part of a longer, proline-rich region (120 residues long, out of which 34 are prolines), predicted as unstructured. It seems therefore, that this entire region should be able to span the width of the periplasm, estimated as 100–200 Å and to reach the outer membrane protein, HasR. Similar flexibility and the lack of regular secondary structure have also been observed for the first several dozen residues of the C-terminal domains of TonB and TolA studied by NMR [40], [41], [42].

The TonB box is the signature of the TBDT. It is required for the transport of substrates and also represents the main region of interaction in the known structures of the complexes TBDT-TonB_CTD_. Predicted as unstructured in the free TBDT, it forms a β-strand upon TonB interaction. To define how HasB_CTD_ interacts with the HasR TonB box, a dodecamer peptide (A_98_LDSLTVLGAGG) was chosen, believed to contain the TonB box motif (underlined) as usually defined by sequence homology [43], [6]. Its size of 12 residues seemed to be sufficient for an interaction with HasB_CTD_ since several peptides of 10 residues, corresponding to the TonB boxes of various transporters have been shown to interact with TonB_CTD_ of *E. coli* [39]. However, this peptide did not reveal any interaction with HasB_CTD_. A second attempt, using a longer 21-mer peptide turned out to be fruitful. This longer peptide corresponds to the whole region of HasR located between the signaling N-terminal domain and the structured part of the plug. Although unstructured in free form, it forms an intermolecular four-stranded β-sheet involving the HasB_CTD_ β3 and the residues G_95_ALALDSL_102_ of the peptide in our model of the complex. The first three residues of β4, formed by the peptide, were absent in the shorter peptide and this difference could explain why it did not interact with HasB_CTD_. However, the same peptide was able to bind to TonB_CTD_ at the same range of concentration as that used with HasB_CTD_. It would thus appear that the TonB box region of HasR interacting with HasB_CTD_ or the “HasB box” is shifted compared to that recognized by TonB.

This shift is also observed when we compare our model of the complex with that of known TonB-TBDT complexes [6], [7]. In our model, the TonB box adopts a β-strand conformation, positioned parallel to the β3 of HasB_CTD_. Residues of the α-helices H1 and H2 also participate in the interaction with the peptide likewise the equivalent regions of two TonB-TBDT complexes. An intermolecular ionic interaction between R38 of HasB_CTD_ and D100 of HasR is observed in our complex, a similar interaction also exists in the BtuB-TonB complex and occurs between D6 of BtuB and R158 of TonB. However, as shown in Figure 5B and 5C, the equivalent of R158 in HasB_CTD_ is not R38 but either R47 (sequentially) or R50 (structurally). It seems that the presence of the helix H1 allows to optimally place R38 in the opening of the groove formed by the α-helices H1, H2 and the strand β3, making in the same time both residues R47 and R50 hardly accessible without any structural rearrangements.

In order to determine the contribution of R38 of HasB_CTD_ to the interaction with the HasB box peptide and validate our model of complex we have mutated this residue to alanine and glutamate and studied the behavior of each of the mutant protein with either the peptide or HasR. Using NMR, we verified that the overall fold of the protein and especially the helix H1 are conserved in both mutants. While the mutation of R38 of HasB to E abolishes the interaction of HasB_CTD_ with the peptide, that of R38A maintains its capacity to bind it. Concerning the interaction with the entire HasR, the variation of ΔH values measured by ITC data revealed that the mutation of R38 significantly decreases the contribution of polar processes. This decrease is more important with the R38E mutation than with R38A.

The ΔH value of interaction of HasR with HasBR38E (ΔH = −7.8±0.2 kcal. mol^−1^) is very similar to that observed in our previous work with HasB_CTD_ and a mutant of HasR with removed stretch of residues D_100_SLTVLGA (ΔH = −7.5±0.1 kcal. mol^−1^). This HasR mutant lacks the residue D_100_, the partner of R38, but also the following residues, which are involved in the formation of the β strand. However in both cases, when either HasB R38 or its partner in HasR, the residue D100 are absent, the variation of ΔH values does not correspond to the loss of only one ionic interaction (reported as 2.5–7 kcal.mol^−1^) but shows the destabilization of the network of polar interactions in the complex formation. Thus this ionic interaction appears to be the key of the complex formation.

The understanding of the mechanism of action of the TonB proteins is an important stage in the development of therapeutic strategies since these proteins are tightly linked to bacterial viability and virulence. The structure of HasB and our result about its interaction with the HasR receptor will help to unravel the mechanism of the active transport of nutrients in bacteria.

## Supporting Information

Figure S1 **ITC analysis of the interaction of TonB_CTD_ with the dodecamer peptide.** Representative experiment is shown. The heat signal (top) is presented together with the binding isotherm derived from the signal (bottom).
(TIF)

Figure S2 **Topology cartoons representing HasB_CTD_ (2M2K) and its structural neighbors, TolA (1LR0) and TonB (1XX3).** The figure was drawn using TopDraw.
(TIF)

Text S1 (DOCX)
