# Supplementary figures and images for: The Structure of HasB Reveals a New Class of TonB Protein Fold
Source: PLoS One. 2013 Mar 19;8(3):e58964. doi: 10.1371/journal.pone.0058964 (PMC3602595; doi:10.1371/journal.pone.0058964)

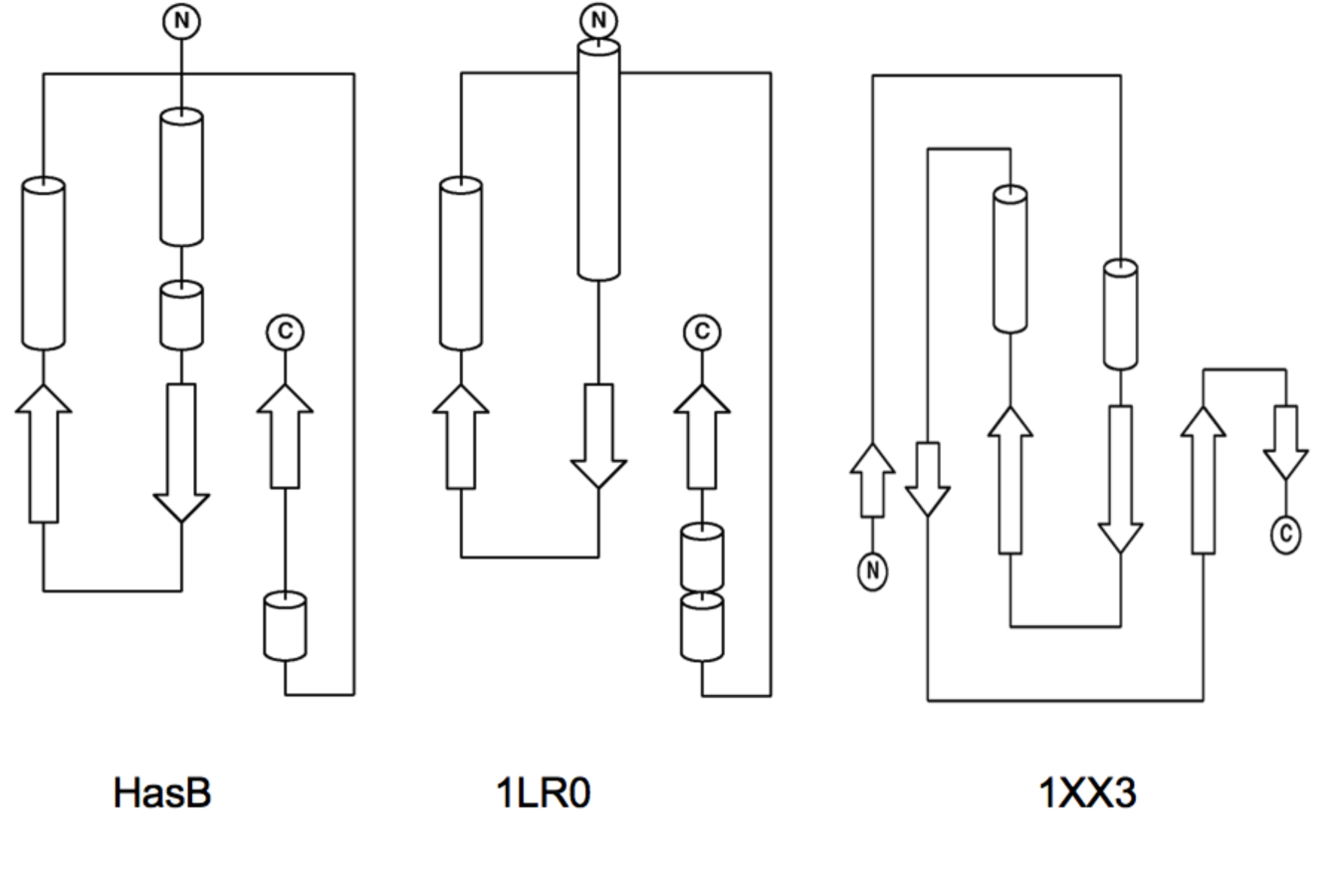

Supplement: Figure S2 — Topology cartoons representing HasBCTD (2M2K) and its structural neighbors, TolA (1LR0) and TonB (1XX3). The figure was drawn using TopDraw. (TIF) [file pone.0058964.s002.tif]
