# Supplementary material for: The Structure of HasB Reveals a New Class of TonB Protein Fold
Source: PLoS One. 2013 Mar 19;8(3):e58964. doi: 10.1371/journal.pone.0058964 (PMC3602595; doi:10.1371/journal.pone.0058964)
Supplement: Text S1 — (DOCX) [file pone.0058964.s003.docx]

**Supplementary Material**

***Docking of the complex structure***

Docking in HADDOCK is driven by the information about the interface region of the complex components. This information can be obtained experimentally (mutagenesis, mass spectrometry, NMR) or using predictive bioinformatics tools, and is introduced into the program as the list of active (residues that are known to make contact within the complex) and passive residues (those that potentially make contact). On the basis of these residues HADDOCK generates an ensemble of ambiguous interaction restraints (AIRs). An AIR is defined between a given active residue and all active and passive residues on the partner molecule. This information is used *via* a dedicated potential energy term to drive the docking process and limit the conformational search problem (as compared to other ab-initio docking programs).

In our case, the input data submitted to HADDOCK consisted of 11 active and 7 passive HasB_CTD_ residues derived from the chemical shift perturbation experiments and from taking into account their solvent accessible surface. For the peptide, 12 active (the “main core” residues 96-107) and 5 passive (remaining “peripheral” residues) residues were given. From these residues HADDOCK generated a set of 23 AIRs, to which the three NOE distance restraints were added.
